# Neonatal anemia due to spontaneous fetomaternal hemorrhage in a term neonate: a case report of an incidental finding and brief review

**DOI:** 10.3389/fped.2025.1625557

**Published:** 2025-09-22

**Authors:** Xinxin Hao, Yuyu Zhang, Chang Gao, Qiushi Wang

**Affiliations:** ^1^ Department of Blood Transfusion, Shengjing Hospital of China Medical University, Shenyang, Liaoning, China; ^2^ Immunohematology Reference Laboratory, Shanghai Blood Center, Shanghai, China

**Keywords:** fetomaternal hemorrhage, neonatal anemia, flow cytometry, blood group serology, Rh incompatibility, perinatal mortality

## Abstract

Fetomaternal hemorrhage (FMH) is a rare perinatal condition characterized by the transplacental transfer of fetal erythrocytes into the maternal circulation. It poses significant risks, including fetal anemia, hydrops fetalis, and maternal alloimmunization, and often remains underdiagnosed due to non-specific clinical presentations and limited diagnostic accessibility. We present a case of a term neonate delivered via cesarean section at 38 weeks’ gestation who exhibited severe anemia and hyperbilirubinemia. Blood group analysis revealed maternal–neonatal Rh incompatibility, while flow cytometry quantified 2.0% fetal erythrocytes in maternal blood. This case underscores the diagnostic value of integrating blood group serology and flow cytometry in FMH confirmation, particularly when standard tests yield equivocal results. A multidisciplinary evaluation of the fetus is the key to early diagnosis and intervention to improve the clinical outcome of perinatal infants.

## Background

Fetomaternal hemorrhage (FMH) is a rare perinatal complication characterized by the transplacental transfer of fetal erythrocytes into the maternal circulation. It may result in fetal anemia, hypoxia, hydrops fetalis, and maternal hemolytic transfusion reactions (1). Its pathogenesis typically involves placental barrier disruption or villous space compromise during gestation or parturition. Clinical manifestations vary depending on hemorrhage volume, with reported incidence ranging from 0.3 to 1 per 1,000 pregnancies (2, 3). Despite its clinical significance, FMH remains underdiagnosed due to non-specific presentations and limitations of detection modalities (4–6). Approximately 40% of pregnancy-related trauma cases are complicated by FMH (7), although other risk factors include placental abnormalities (e.g., abruption and chorioamnionitis), maternal comorbidities (e.g., gestational hypertension), invasive procedures (e.g., amniocentesis), and umbilical cord anomalies (4, 8). Notably, many FMH cases occur in otherwise uncomplicated pregnancies (9). This condition should be considered in the differential diagnoses of severe neonatal anemia, non-immune hydrops fetalis, and unexplained stillbirth. We present a term neonate with severe anemia secondary to FMH following cesarean delivery, highlighting diagnostic approaches and management strategies.

## Case presentation

A neonate (birth weight 2,655 g) was delivered via cesarean section at 38 weeks’ gestation to a gravida 2 para 1 mother with hepatitis B virus carrier status managed with tenofovir disoproxil fumarate. Hepatitis B immunoglobulin was administered to the neonate. The mother had no history of blood transfusion or abdominal trauma during pregnancy. Apgar scores were 9 and 10 at 1 and 5 min postpartum. Physical examination revealed pallor, scattered petechiae, and SpO_2_ of 95%, with no other abnormalities on physical examination post-delivery. Laboratory investigations revealed: hemoglobin 85 g/L (reference:180–190 g/L), hematocrit 29.98% (37%–47%), platelets 167 × 10^9^/L (135–350 × 10^9^/L), reticulocytes 19.5% (0.5%–2%), WBC 9.54 × 10^9^/L (15–20 × 10^9^/L), prothrombin time (PT) 10.2 s (9.4–12.5 s), activated partial thromboplastin time (APTT) 35 s (21–37 s), antithrombin (AT) 72% (83%–128%), and total protein 48.9 g/L (60–83 g/L), albumin 32.0 g/L (35–53 g/L), globulin 16.90 g/L (20–40 g/L), alanine aminotransferase (ALT) 17 U/L (0–40 U/L), aspartate aminotransferase (AST) 134 U/L (5–34 U/L), hyperbilirubinemia [total bilirubin 46.1 µmol/L (3.4–20.5 µmol/L], and unconjugated bilirubin 39.7 µmol/L (3.4–11.9 µmol/L).

**Figure 1:**
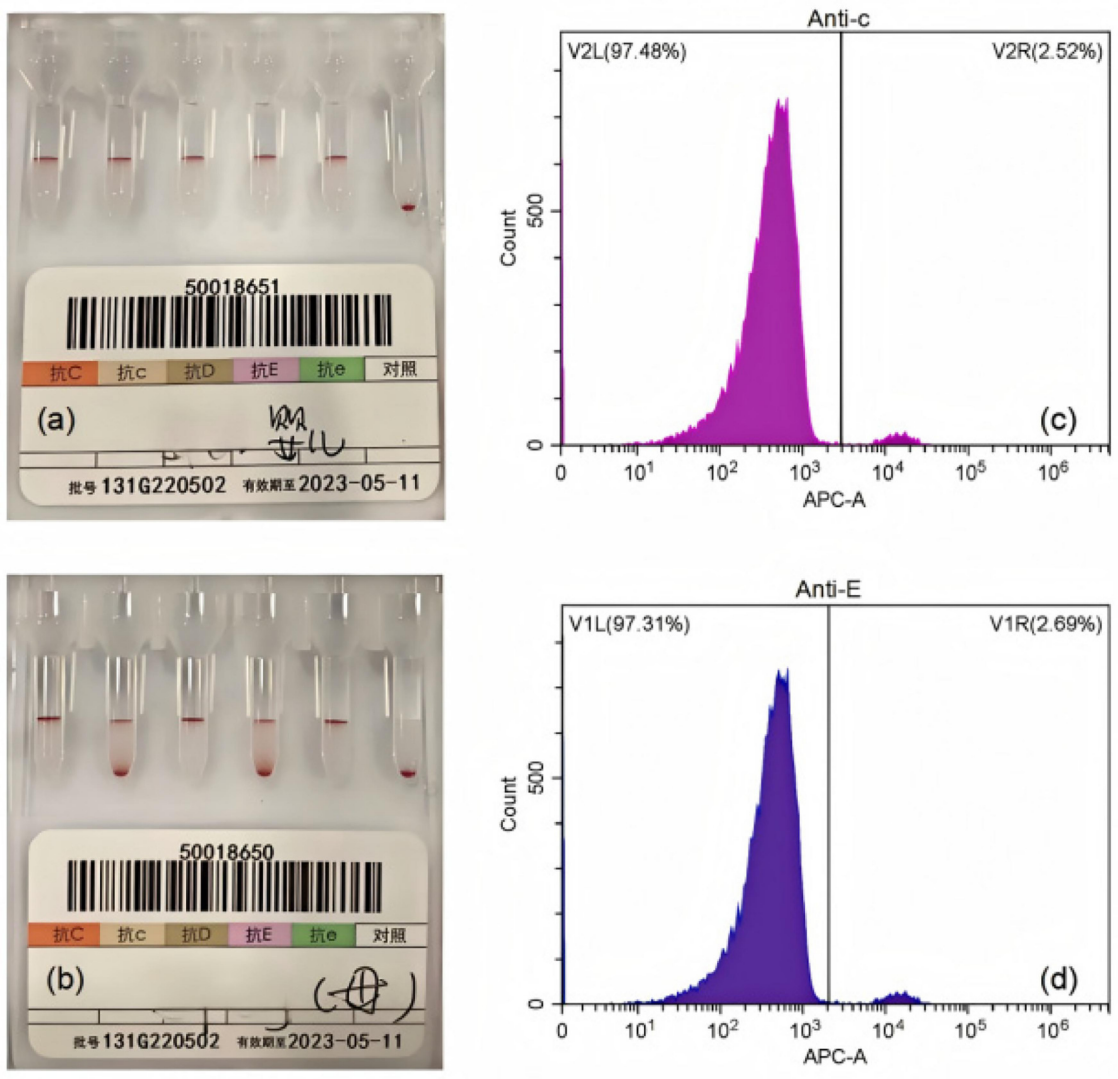
Representative laboratory findings in the diagnosis of FMH. **(a)** Rh phenotyping of the newborn. **(b)** Rh phenotyping of the mother before capillary centrifugation. **(c-d)** Flow cytometric plots of maternal blood labeled with anti-c **(c)** and anti-E **(d)** antibodies.

Initial evaluation excluded infectious (normal C-reactive protein, absence of thrombocytopenia/acidosis) and nutritional etiologies (normal erythropoietin, ferritin, folate, and B_12_ levels). Hemolytic workup revealed maternal O RhD+/neonatal O RhD+ compatibility, a negative direct antiglobulin test, and absence of irregular antibodies. Rh phenotyping identified maternal CCDee [dual population(DP) of c and E antigens] and neonatal CcDEe profiles after capillary centrifugation (Figures 1a,b). Flow cytometric analysis using anti-E/c monoclonal antibodies detected 2.0% fetal erythrocytes in the maternal circulation (≈44 mL fetomaternal transfusion) (Figures 1c,d), corroborated by antenatal ultrasound findings of placental thickening at 35 weeks. The newborn received red blood cell transfusions (26 mL on Day 3 and 40 mL on Day 4) for anemia correction, demonstrating hemoglobin recovery to 112 g/L by Day 7, permitting discharge.

Figure 1 presents representative laboratory findings in the diagnosis of FMH. The Rh blood group was determined using the microcolumn gel method. Flow cytometry was used to assess fetal blood volume in the maternal circulation (Figure 1a). Rh phenotyping of the newborn (CcDEe) was performed using the microcolumn gel assay (Figure 1b). Rh phenotyping of the mother (CCDee) demonstrating DP patterns before capillary centrifugation (Figures 1c,d). Flow cytometric plots of maternal blood labeled with anti-c (Figure 1c) and anti-E (Figure 1d) antibodies confirmed the presence of fetal erythrocytes.

## Discussion and conclusions

The diagnosis of FMH requires a high index of clinical suspicion and a multimodal assessment. Antenatal diagnosis remains challenging except in trauma-induced cases, underscoring the need for obstetric vigilance in cases of reduced fetal movements and sinusoidal fetal heart rate patterns (10). Laboratory methods include the following: Rosette test (for RhD incompatibility), Kleihauer–Betke acid elution test (KB test; FMH screening), flow cytometry (quantifying fetal RBCs via anti-HbF/anti-D antibodies), and abnormal elevation of maternal alpha-fetoprotein (AFP) (4, 14, 15). Ultrasonographic markers—such as placental intervillous thrombi and middle cerebral artery peak systolic velocity (MCA-PSV) >1.5 MoM—aid in severity stratification (11, 12). These approaches facilitate FMH identification and guide subsequent management. In complex pregnancies (e.g., ≥35 weeks’ gestation or multiples), continuous fetal heart monitoring offers a straightforward approach, enabling prompt delivery upon detection of reduced fetal movement (13). In this case, ultrasound revealed placental thickening at 35 weeks of gestation. However, beyond intensified fetal monitoring, no further diagnostic evaluation was pursued. Focal placental thickening warrants clinical attention, as it may indicate existing fetal anemia—a possibility that was overlooked in this instance due to the absence of overt maternal triggers and non-specific symptoms. Potential etiologies of placental thickening include pregnancy complications (e.g., hypertensive disorders, diabetes), intrauterine infections, fetal abnormalities (e.g., anemia and growth restriction), and primary placental pathologies (16, 17). Such findings necessitate comprehensive clinical evaluation and evidence-based obstetric management. Although no adverse outcomes occurred in this case, a conservative approach may delay diagnosis, increase perinatal healthcare costs, and heighten familial psychological burden. While expectant management is common for idiopathic placental thickening, obstetricians detecting placental thickening with potential fetal anemia should recommend KB testing, MCA-PSV, AFP, or even parental blood typing for differential diagnosis.

Postnatal confirmation relies on fetal RBC quantification via flow cytometry or blood group serology. In this case, maternal (CCDee) and neonatal (CcDEe) Rh type discordance prompted flow cytometry using anti-E and anti-c antibodies (replacing conventional anti-HbF), enhancing diagnostic precision. Nevertheless, flow cytometry remains underutilized in resource-limited settings (18). Placental examination in FMH may reveal immature villous development, chorioedema, intervillous thrombi, and nucleated red blood cells in fetal blood vessels—features that support the diagnosis (19). Critically, 14% of perinatal deaths are attributed to undiagnosed FMH (20), necessitating systematic evaluation of unexplained neonatal anemia.

**Figure 2:**
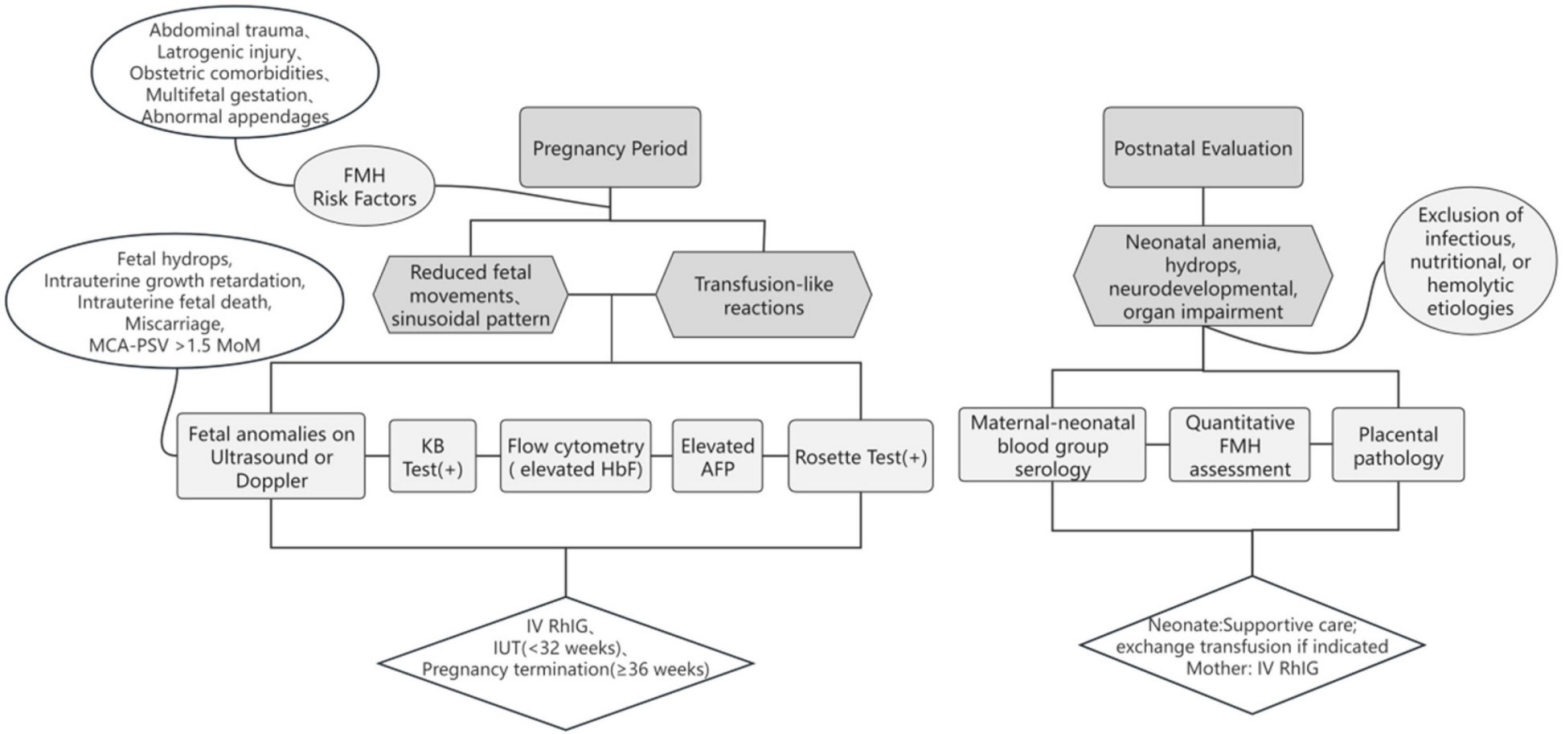
Clinical diagnosis and management for FMH in the perinatal period.

Management depends on gestational age and hemorrhage volume [>30 mL diagnostic threshold (21); >80 mL or 20% fetal blood volume associated with high mortality (8, 22)]. Intrauterine transfusion is recommended before 32 weeks’ gestation, while prompt delivery is preferred beyond 36 weeks to optimize outcomes. Although most FMHs are non-recurrent, prior FMH with maternal–fetal blood group incompatibility may precipitate hemolytic disease of the fetus and newborn (HDFN) or other sequelae. Obstetricians should emphasize prenatal FMH history assessment and optimize maternal management. While anti-D prophylaxis is standard for RhD-negative mothers, consensus is lacking for non-RhD alloimmunization (23). A simple process for clinicians to identify and manage FMH is shown in Figure 2.

FMH remains a key but underrecognized cause of perinatal morbidity and mortality. This case highlights the importance of maintaining a high level of clinical suspicion of FMH in newborns with unexplained anemia, especially when routine causes are excluded, and demonstrates the need for multidisciplinary collaboration and standardized diagnostic protocols.

## Data Availability

The original contributions presented in the study are included in the article/Supplementary Material, further inquiries can be directed to the corresponding author.
